# Comprehensive bioinformatics analysis reveals the crosstalk genes and immune relationship between the systemic lupus erythematosus and venous thromboembolism

**DOI:** 10.3389/fimmu.2023.1196064

**Published:** 2023-07-03

**Authors:** Jingfan Yu, Jian Yang, Qifan He, Zhixuan Zhang, Guoxiong Xu

**Affiliations:** ^1^ Department of Vascular Surgery and Intervention, The Affiliated Suzhou Hospital of Nanjing Medical University, Suzhou Municipal Hospital, Suzhou, Jiangsu, China; ^2^ Department of Interventional Radiology, The First Affiliated Hospital of Soochow University, Suzhou, China

**Keywords:** venous thromboembolism, systemic lupus erythematosus, bioinformatics analysis, transcriptomics, immune cells infiltration, unsupervised clustering

## Abstract

**Background:**

It is well known that patients with systemic lupus erythematosus (SLE) had a high risk of venous thromboembolism (VTE). This study aimed to identify the crosstalk genes between SLE and VTE and explored their clinical value and molecular mechanism initially.

**Methods:**

We downloaded microarray datasets of SLE and VTE from the Gene Expression Omnibus (GEO) dataset. Differential expression analysis was applied to identify the crosstalk genes (CGs). Gene Ontology (GO) and Kyoto Encyclopedia of Genes and Genomes (KEGG) pathway enrichment analyses were performed on the shared genes. The shared diagnostic biomarkers of the two diseases were further screened from CGs using least absolute shrinkage and selection operator (Lasso) regression. Two risk scores for SLE and VTE were constructed separately to predict the likelihood of illness according to the diagnostic biomarkers using a logical regression algorithm. The immune infiltration levels of SEL and VTE were estimated *via* the CIBERSORT algorithm and the relationship of CGs with immune cell infiltration was investigated. Finally, we explored potential phenotype subgroups in SLE and VTE based on the expression level of CGs through the consensus clustering method and studied immune cell infiltration in different subtypes.

**Result:**

A total of 171 CGs were obtained by the intersection of differentially expressed genes (DEGs) between SLE and VTE cohorts. The functional enrichment shown these CGs were mainly related to immune pathways. After screening by lasso regression, we found that three hub CGs (RSAD2, HSP90AB1, and FPR2) were the optimal shared diagnostic biomarkers for SLE and VTE. Based on the expression level of RSAD2 and HSP90AB1, two risk prediction models for SLE and VTE were built by multifactor logistic regression and systemically validated in internal and external validation datasets. The immune infiltration results revealed that CGs were highly correlated with multiple infiltrated immunocytes. Consensus clustering was used to respectively regroup SLE and VTE patients into C1 and C2 clusters based on the CGs expression profile. The levels of immune cell infiltration and immune activation were higher in C1 than in C2 subtypes.

**Conclusion:**

In our study, we further screen out diagnostic biomarkers from crosstalk genes SLE and VTE and built two risk scores. Our findings reveal a close relationship between CGs and the immune microenvironment of diseases. This provides clues for further exploring the common mechanism and interaction between the two diseases.

## Introduction

Systemic lupus erythematosus (SLE) is a chronic autoimmune disease with multisystemic involvement and complicated clinical symptoms(1). It should be noted that SLE patients may increase the risk of venous thromboembolism (VTE) according to past research (2–4). Abnormality of the blood coagulation system is also a significant characteristic of SLE patients (5). VTE comprises both pulmonary embolism (PE) and deep vein thrombosis (DVT) with a high incidence rate. According to estimates, approximately 1 or 2 cases per 1000 persons per year in USA (6). Accumulating evidence suggested the pathogenesis of VTE is not restricted to the coagulation system only, but the immune system is also a crucial link for the formation of thrombosis (7). Moderate and severe inflammation was found in 13.4% and 1.3% of thrombus samples from pulmonary thromboendarterectomy (8). Gene microarray analysis of PE and DVT specimens presented nearly 10% of differential expression genes were immunity/inflammatory genes (9).

The coagulation and immune systems have a common evolutionary origin (10). The relationship between immunity and plasma coagulation is an intricate and interconnected network. Especially, aberrant interactions among immune cells play a critical role at the crossroads between inflammation and haemostasis (11). For example, platelets can promote neutrophil activation and lead to neutrophil extracellular traps (NETs) (12). These aggregates of decondensed chromatin concentrate high amounts of crucial autoantigens for the development of SLE and coagulation triggers such as TF or von Willebrand factor (13). TF pathway-dependent thrombin formation is an indispensable part of the process of thrombosis. Some studies have shown that TF pathway activation can be detected in patients with SLE (14).

However, although an enormous amount of research implies the intricate connection between immunity and coagulation, the relevance of VTE and SLE were still unclear. Thus, we first used multiple bioinformatics techniques to comprehensively analyze the relationship between SLE and VTE based on microarray and high-throughput sequencing and explore potential cellular and molecular mechanisms. In this study, we identified the potential crosstalk genes (CGs) between SLE and VTE and evaluated the interaction between these CGs and infiltrating immune cells utilizing a variety of advanced statistical algorithms to gain a deeper understanding of the pathophysiological processes that may link SLE and VTE. Moreover, the latent value of CGs in disease diagnosis was assessed and validated in different cohorts.

## Materials and methods

### Data download

We obtained the gene microarray data of SLE and VTE, which was downloaded from the GEO database (https://www.ncbi.nlm.nih.gov/geo/) (15). Based on the GPL570 platform, the GSE61635 dataset contains 79 blood samples of SLE patients and 30 healthy controls. To estimate the diagnostic efficiency, the GSE50772 dataset based on GPL570 was downloaded, containing 81 SLE blood samples and health controls. A gene expression dataset related to VTE (GSE19151) was based on the GPL571 platform and included 133 blood samples (70 VTE patients and 63 healthy controls). To estimate the diagnostic efficiency, we also downloaded GSE48000 (based on the GPL10558 platform), containing 134 VTE samples and 44 healthy controls. All samples were taken from whole blood and all patients had been comprehensively diagnosed by pathologic biopsy, blood examinations and imaging examination. The raw CEL files downloading from GEO datasets were normalized by the robust multichip average (RMA) which was implemented in the R package affy (version 1.54.0) (16). When a gene symbol corresponded to multiple probes, the mean expression level of all probes served as the final value.

### Identification of CGs and enrichment analyses

The ‘limma’ R package was used to screen the differentially expressed genes (DEGs) from the GSE61635 and GSE19151 datasets. The selection criteria of DEGs in GSE61635 were set as |log FC| ≥ 1 and *p*-value> 0.05, and the DEGs were screened for GSE19151 with and |log FC| ≥ 0.8 and p-value> 0.05. The results were displayed utilizing gene clustering heatmaps and volcano maps. A combined analysis of DEGs between GSE61635 and GSE19151 was conducted by drawing Venn diagrams. Overlapping genes were considered the crosstalk genes (CGs) of the two diseases and were extracted for further functional enrichment analysis. The Gene Ontology (GO) enrichment analysis was conducted by the R package ‘clusterProfiler’ (17). The significant differential GO terms were defined with a strict cut-off of *p* < 0.01. The gene set variation analysis (GSVA) was performed to calculate the normalized Enrichment score (NES) of the hallmark gene set (c2.cp.kegg.v7.2) using the ‘GSVA’ R package, and *p*-value < 0.05 and FDR < 0.25 were considered to be statistically significant (18). Gene set enrichment analysis (GSEA) was also implemented to identify the biological attribute and gene function by R package ‘clusterProfiler’, and *p*-value < 0.05 and FDR < 0.25 were considered to be the statistically significant difference (19).

### Selection of shared diagnostic CGs and establishment of risk scores for VTE and SLE

Lasso regression was employed to identify the potential diagnostic CGs for VTE and SLE using the ‘glmnet’ package of R software (20). The optimal values of the penalty parameter were determined through 10 cross-validations. Subsequently, we selected overlapping CGs as optimal shared diagnostic CGs and assess their expression levels in the several cohorts. The area under the curve (AUC) of receiver operating characteristic (ROC) was utilized to evaluate the diagnostic effectiveness of these biomarkers. The correlation analyses among the shared diagnostic CGs were applied to avoid the multicollinearity problem of variables. Multi-factor logical regression was performed to establish the SLE (GSE61635) and VTE (GSE19151) risk scores based on the shared diagnostic biomarkers. These two predictive scores for each sample were calculated by the expression of shared diagnostic CGs and their logical regression coefficient. The risk score formula was established as follows:


$$Risk\;score=\sum_{i}Coefficient\;of\;(i)\times Expression\;of\;gene\;(i)$$

The coefficient of the gene (i) is the regression coefficient of the gene (i), and the Expression of the gene (i) is the expression value of gene (i) for each patient. Nomograms were drawn by the ‘rms’ package to elevate the operability and practicability of the risk models. In the internal (GSE61635 and GSE19151) and external (GSE50772 and GSE48000) validation analyses, the ROC curve and AUC value assessed the efficiency of diseases prediction for the risk models, and the calibration curves and C-indexes were used to evaluate the consistency of prediction and actual observation. Additionally, we used ‘ComBat’ function in ‘sva’ package (2) to remove the batch effect between GSE19151 and GSE48000 and merge them into one dataset (VTE combined database). The same approach was then used to merge the SLE datasets (GSE61635 and GSE50772) and validate the prediction of risk scores in these combined datasets.

### The association networks of CGs

The protein-protein interaction network (PPI) of the CGs was downloaded from the STRING online dataset (https://cn.string-db.org/) (21) and visualized using Cytoscape software (22). The minimum required interaction score of association was set as 0.4. The key CGs of the network were identified using a Cytoscape plugin named cytoHubba which contains several topological algorithms such as Maximum Neighborhood Component (MNC), maximal clique centrality (MCC), Edge Percolated Component (EPC), Degree, and so on (23). For further checking the expression features for crucial genes in different datasets, we respectively compared their expression levels based on the VTE cohort (GSE19151 and GSE48000) and SLE cohorts (GSE61635 and GSE50772) using the t.test algorithm.

### Immune infiltration analyses of the SLE and VTE cohorts

The distribution of immune cells between diseases and normal groups was explored using the CIBERSORT algorithm, which is a tool to calculate the relative percentage of 24 immune cells based on gene expression matrix (24). The immunocyte types with low infiltration levels (mean value < 1%) were eliminated. The ‘pheatmap’ package was used to draw heatmaps that can visualize the correlation of five hub CGs with the abundance of immune cells.

### Detection of CGs-related subsets

The unsupervised consensus clustering method (K-means) was applied to identify CGs-related subtypes in SLE and VTE patients. The unsupervised clustering “Pam” method based on Euclidean and Ward’s linkage was carried out to process this analysis, executed by using the “ConsensuClusterPlus” R package and repeated 1,000 times to ensure classification stability (25). The package “MCPcounter” was used to estimate the absolute population abundance of tissue-infiltrating immunocytes from transcriptomic data (26). Then, we assessed the distribution of SLE and VTE subtypes in immunocytes infiltration data of MCPcounter and CIBERSORT. Finally, we compared the GSVA score between different subtypes using the limma package and displayed the remarkably different pathways by heatmaps. Fifty hallmark gene sets were curated from the MSigDB as the reference set.

### Statistical analysis

R software 3.6.5 was performed for statistical analyses and visualization. For differences of gene expression levels or immunocyte fractions between different clinical groups were analyzed by a two-sided Wilcoxon test. Correlation analysis was conducted using the Spearman test. The *p*-value was adjusted by the FDR method for multiple hypothesis testing. Dichotomous variables were compared using the chi-square test.

## Results

### Identification of CGs in VTE and SLE cohorts

**Figure 1:**
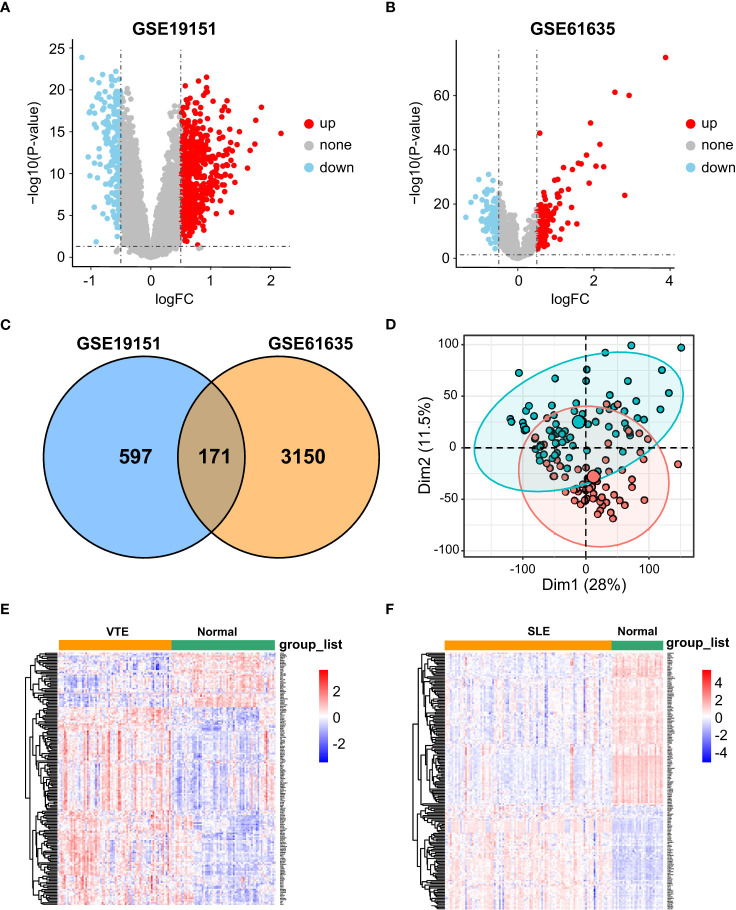
Differential expression gene analysis. **(A, B)** Volcano plots showed differentially expressed genes (DEGs) in GSE19151 and GSE61635. **(C)** Venn plots of the crosstalk genes (CGs) between GSE19151 and GSE61635. **(D)** The distribution characteristics of samples based on PCA results in GSE19151. **(E, F)** The expression pattern of CGs in GSE61635 and GSE19151.

In the SLE dataset GSE61635, a total of 3321 DEGs, consisting of 2492 upregulated DEGs and 829 downregulated DEGs, were identified (**Figure 1A**). In the VTE dataset GSE19151, a total of 768 DEGs, consisting of 421 upregulated DEGs and 347 downregulated DEGs, were identified (**Figure 1B**). As the Venn diagram showed in **Figure 1C**, there were 171 overlapping CGs between SLE and VTE cohorts. PCA analysis of the expression matrix suggested that samples in the disease and control groups were clearly distributed on both sides (**Figures 1D**, **S1** **)**. The heatmaps show the expression pattern of CGs in VTE and SLE cohorts (**Figures 1E, F**).

### Enrichment analysis of CGs

**Figure 2:**
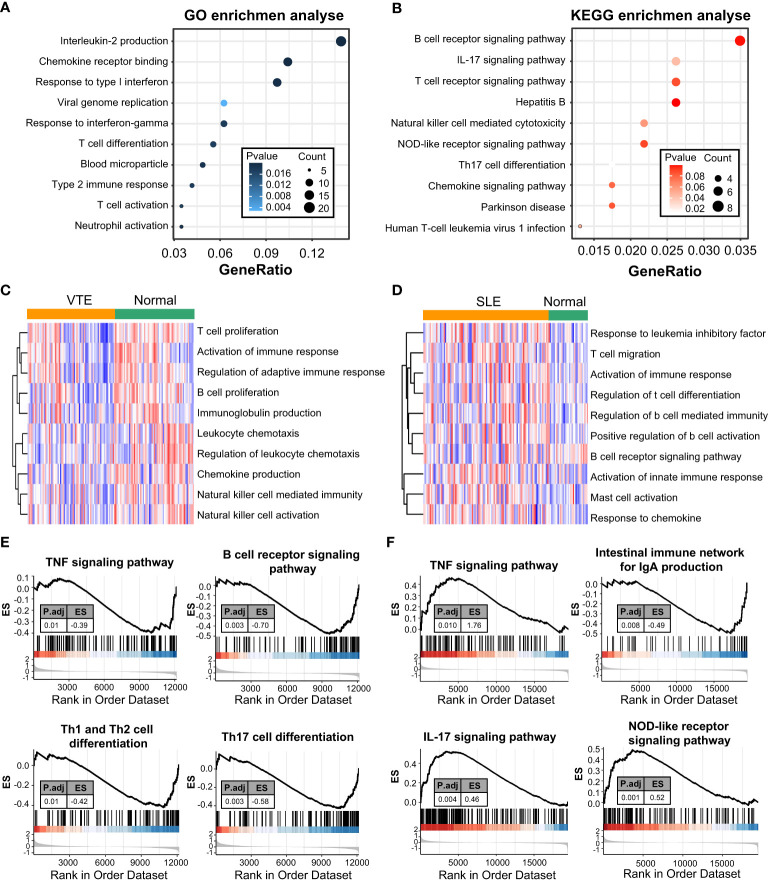
Function enrichment and pathway enrichment analysis. **(A, B)** GO and KEGG enrichment analyses of CGs. **(C, D)** GSVA analyses in GSE19151 and GSE61635. **(E, F)** GSEA analyses base on GSE19151 and GSE61635.

GO and KEGG enrichment analyses were conducted to investigate the biological function of CGs. The results indicated that CGs were mainly enriched in immune and inflammatory pathways, including the B/T cell receptor signaling pathway, Interleukin-2 production, T cell differentiation, T cell activation and chemokine signaling pathway (**Figures 2A, B**). Besides, the results of GSVA of VTE cohorts showed that immunity and inflammation pathways, such as regulation of adaptive immune response, activation of the immune response, chemokine production, and natural killer cell-mediated immunity, were mainly enriched in the normal group compared with the VTE group (**Figure 2C**). The results of GSVA of SLE cohorts suggested that immunity pathways, including positive regulation of B cell activation, Activation of innate immune response, Mast cell activation, and response to chemokine, were mostly enriched in the SLE group compared with the normal group (**Figure 2D**). The immune response patterns of the two diseases seem to be different. The GSEA was also applied to evaluate the signaling pathways involved in the CGs. The results demonstrated that the CGs were negatively linked to the immune pathways (TNF signaling pathway, B cell receptor signaling pathway, and Th1/Th2/Th17 cell differentiation) in VTE (**Figure 2E**) and were positively linked to the immune responses (TNF signaling pathway, IL-17 signaling pathway, and NOD-like receptor signaling pathway) in SLE (**Figure 2F**). These results illustrated that CGs were involved in the regulation of immune function in the SLE and VTE.

### Identification of optimal shared diagnostic CGs

**Figure 3:**
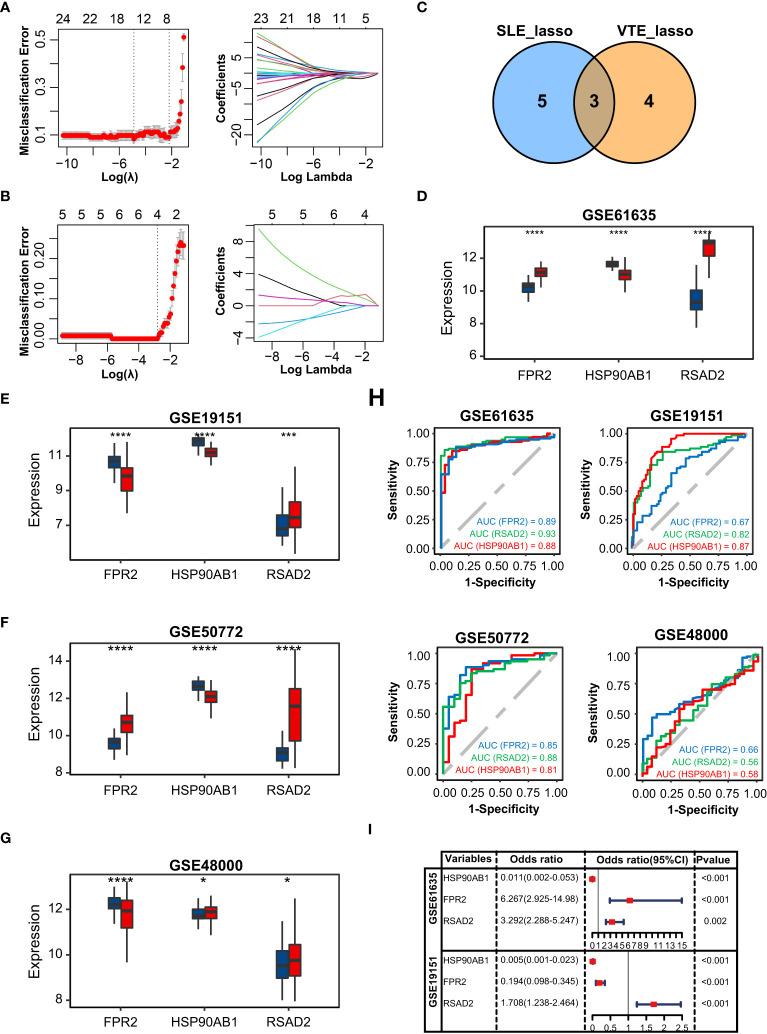
Identification of potential shared diagnostic CGs. **(A, B)** 10-fold cross-validation to select the optimal tuning parameter log (lambda) in the GSE61636 and GSE19151 database. **(C)** Venn diagram showing the optimal diagnostic biomarkers. **(D-G)** The expression level of the shared diagnostic biomarkers in GSE61636, GSE19151, GSE50772 and GSE48000. **(H)** ROC curve of the shared diagnostic CGs in GSE61636, GSE19151, GSE50772 and GSE48000. **(I)** Single-factor logistic regression of the shared diagnostic CGs. Statistical significance at the level of ns ≥ 0.05, * <0.05, *** <0.001 and **** <0.0001.

In GSE61635, the lasso regression algorithm identified eight diagnostic CGs under the most appropriate λ=0.14 (**Figure 3A**). In GSE19151, the lasso regression algorithm identified seven diagnostic CGs under the most appropriate λ=0.061 (**Figure 3B**). Three overlapping CGs (HSP90AB1, FPR2, and RSAD2) were screened to be the optimal shared diagnostic CGs for SLE and VTE (**Figure 3C**). **Figures 3D-G** show the differential gene expression patterns of the three candidate biomarkers in SLE (GSE61635 and GSE50772) and VTE datasets (GSE19151 and GSE48000). Compared with the control group, FPR2 and RSAD2 were upregulated and HSP90AB1 was downregulated in the SLE group. Meanwhile, FPR2 and HSP90AB1 were downregulated and RSAD2 was upregulated in the VTE group. We then used ROC curves to validate the diagnostic efficacy of HSP90AB1, FPR2, and RSAD2 in the SLE dataset and the VTE dataset, and all showed potent performance of disease identification (**Figure 3H**). Univariate logistic analysis of the three candidate biomarkers also showed they can accurately distinguish the patients and healthy individuals (**Figure 3I**).

### Construction and validation of SLE and VTE risk scores

**Figure 4:**
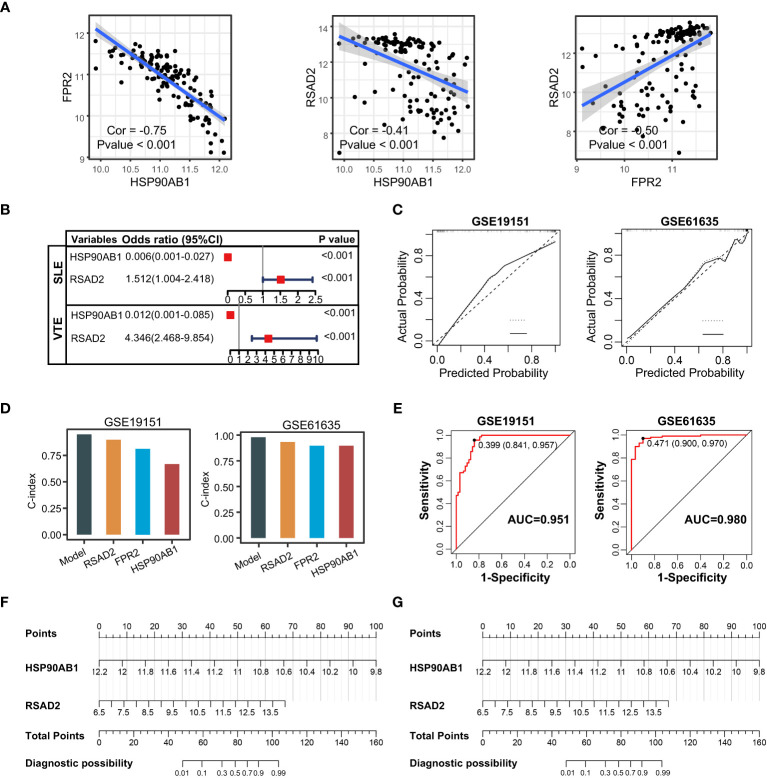
Construction of risk scores. **(A)** The correlation among the shared diagnostic CGs in GSE61635. **(B)** The multivariate logistic regression comprising of HSP90AB1 and RSAD2. **(C)** Calibration curves of the VTE and SLE risk models. **(D)** C-index of risk models and single variables. **(E)** ROC curves of the two risk models. **(F, G)** Nomogram predicting the probability of VTE and SLE. The left template for SLE and the right for VTE.

**Figure 5:**
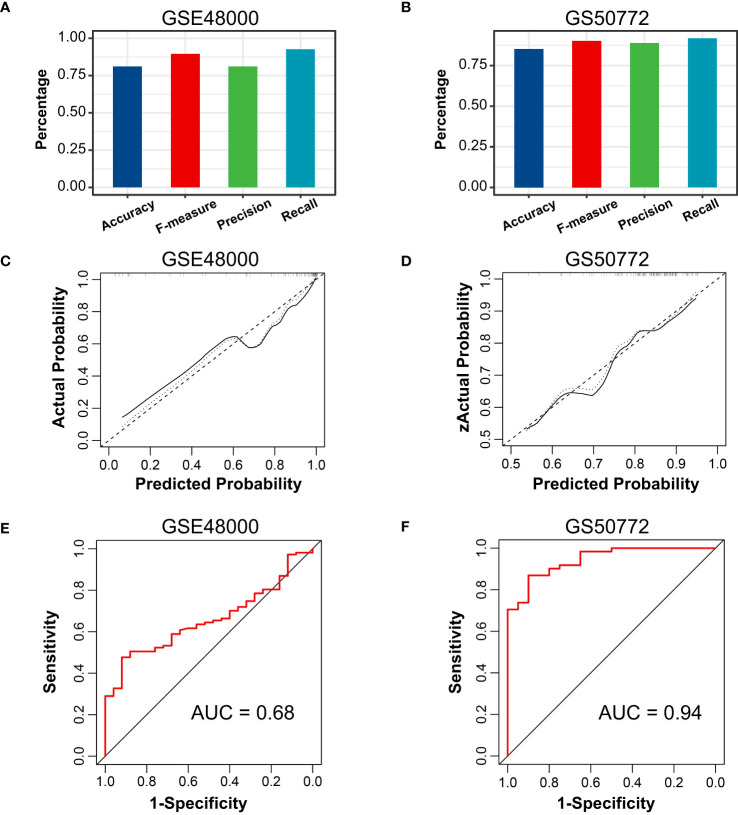
External verification of VTE and SLE risk scores. **(A, B)** The accuracy, precision, recall, and f-measure of VTE and SLE risk models in GSE4800 and GSE50772. **(C, D)** Calibration curves of the VTE and SLE risk scores in the GSE4800 and GSE50772. **(E, F)** ROC curves of the two risk models in the GSE4800 and GSE50772.

The correlation among three variables in GSE61635 and GSE19151 was illustrated in **Figures 4A**, **S2**. The correlation coefficient between the HSP90AB1 and FPR2 was 0.75 in GSE61635. We consider the high possibility of multicollinearity between these two variables. So the FPR2 was removed and HSP90AB1 and RSAD2 were further incorporated into the multivariate logistic regression model to build predictive scores (**Figure 4B**). The results of regression indicated that HSP90AB1 was an independent protective factor and RSAD2 was an independent risk factor both in SLE and VTE. By weighting the normalized expression level of HSP90AB1 and RSAD2 to the regression coefficients of the multivariate logistic regression analysis, we established an SLE risk score model (SLE risk score = normalized expression level of RSAD2 * 1.469 - normalized expression level of HSP90AB1 * 4.389) and VTE risk score model (VTE risk score = normalized expression level of RSAD2 * 0.413 - normalized expression level of HSP90AB1 * 5.184). Both in the SLE and VTE cohorts, the bias-corrected lines in the calibration plot were close to the ideal curve, which indicated good consistency of predictive models (**Figure 4C**). The C-index of the SLE and VTE risk models exceeded that of the single single-factor risk models, suggesting that our risk scores had favorable efficacy for forecasting the diseases (**Figure 4D**). **Figure 4E** shows the predictive potential of the two risk scores using ROC curves. The area under the ROC curve (AUC) of the SLE risk score was 0.98 on the GSE61635 and the VTE risk score was 0.95 on the GSE19151. Two nomograms respectively based on SLE and VTE risk scores were constructed to provide clinicians with a quantitative approach to predicting the risk of illness (**Figures 4F, G**). In addition, the results of external validation analysis also demonstrated the two risk scores achieved excellent predictive performance. The accuracy, precision, recall rate, and f-measure of the two risk scores all exceeded 0.75 in GSE50772 and GSE48000 (**Figures 5A, B**). The calibration curves (**Figures 5C, D**) and ROC curves (**Figures 5E, F**) based on the external data verified their dependable performance to predict disease. The ROC curves of SLE and VTE combined datasets suggested that the two risk scores had excellent performance (**Figure S3**).

### The PPI network of hub CGs

**Figure 6:**
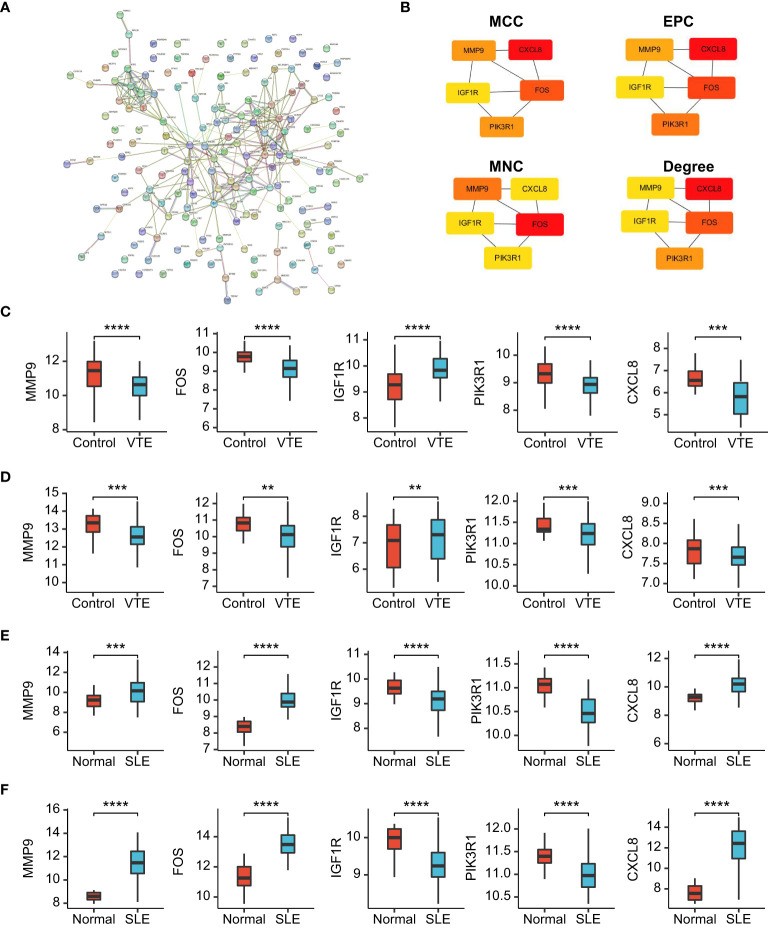
PPI network and gene expression validation analysis. **(A)** PPI network of the CGs. **(B)** The top 5 CGs ordered by the MCC, EPC, MNC and Degree algorithm. **(C-F)** The expression level of hub CGs in GSE19151, GSE48000, GSE61635, and GSE50772. Statistical significance at the level of ns ≥ 0.05, ** <0.01, *** <0.001 and **** <0.0001.

To identify the potential interaction of CGs, we used Cytoscape software to build a PPI network according to the STRING database, integrating 28 nodes and 64 edges (**Figure 6A**). Next, hub genes were extracted from CGs using four different topological analysis methods (MCC, MNC, EPC, and degree). The results of the four algorithms all point to the five hub CGs: MMP9, FOS, IGF1R, PIK3R1, and CXCL8 (**Figure 6B**). Compared with the corresponding control group, these five hub CGs were all significantly up/down-regulated in the external (GSE19151 and GSE61635) and internal datasets (GSE48000 and GSE50772) (**Figures 6C–F**).

### Comparison of the immune microenvironment in VTE and SLE cohorts

**Figure 7:**
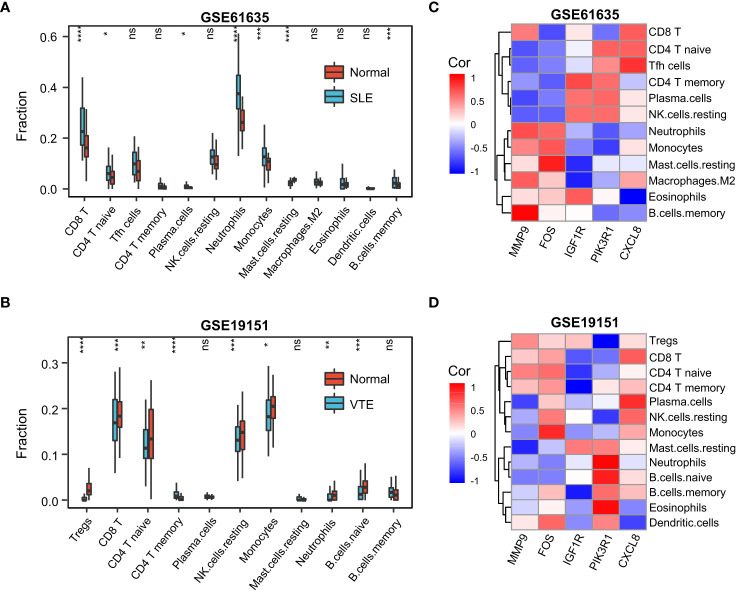
The immune infiltration landscape of SLE and VTE. **(A, B)** Relative immune cell abundance based on CIBERSORT between disease and normal groups in GSE61635 and GSE19151. **(C, D)** The heatmaps showed the correlation of hub CGs with immune cell infiltration. Statistical significance at the level of ns ≥ 0.05, * <0.05, ** <0.01, *** <0.001 and **** <0.0001.

To further explore the immune landscapes in VTE and SLE cohorts, the percentage of the 22 kinds of immune cells in each sample was calculated using the CIBERSORT algorithm. In the GSE61635, the most of immune cells, such as neutrophils, CD8 T cells, naive CD4 T, Monocytes, activating CD4 T memory, resting NK cells, naive B cells, and follicular helper-like T cells, significantly infiltrated in the SLE group rather than normal group (**Figure 7A**). In the GSE19151, the majority of immune cells, such as monocytes, regulatory T cells, activated memory CD4 T cells, CD8 T cells, naive CD4 T cells, naive B cells, resting NK cells, and macrophages, significantly infiltrated in the normal group rather than VTE group (**Figure 7B**). These results suggested that SLE patients exhibit a state of immune activation and VTE patients had a state of immunosuppression. Five hub CGs had a significant correlation with multiple immune cell infiltration levels both in the SLE and VTE cohorts (**Figures 7C, D**).

### Association between infiltrating immunocytes and CGs subtypes

**Figure 8:**
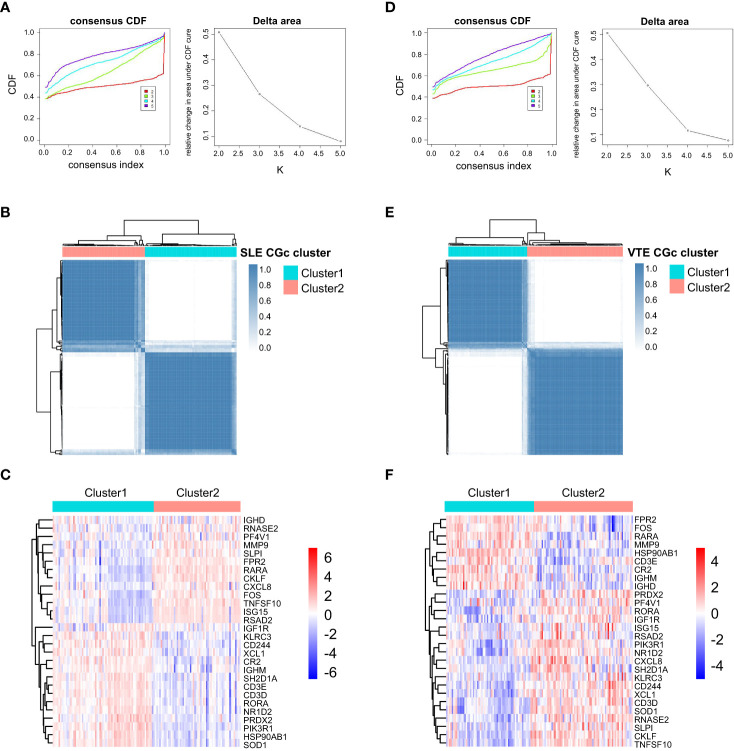
Consensus clustering. **(A)** Consensus CDF when k = 2-5 and Relative alterations in the area under CDF curve based on GSE61635. **(B)** Consensus matrix heatmap of SLE cohorts when k = 2. **(C)** The heatmaps of CGs expression between C1 and C2 clusters of SLE subtypes. **(D)** Consensus CDF when k = 2-5 and relative alterations in the area under CDF curve based on GSE19151. **(E)** Consensus matrix heatmap of VTE cohorts when k = 2. **(F)** The heatmaps of CGs expression between C1 and C2 clusters of VTE subtypes.

**Figure 9:**
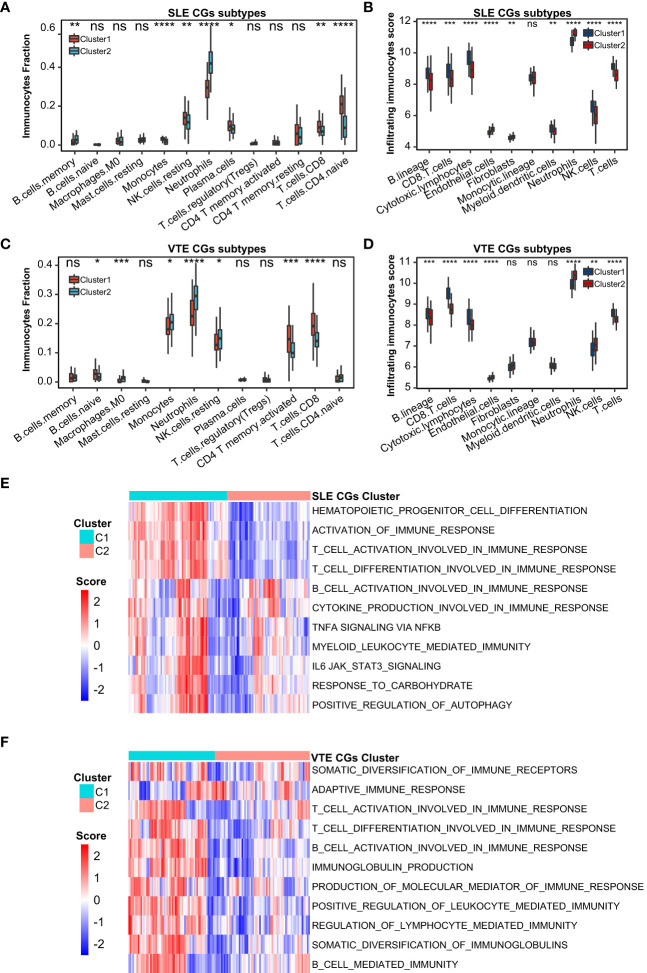
Correlation of two CGs subtypes and immune cell infiltration. **(A, B)** The immune cell distribution in C1 and C2 of SLE CGs subtypes based on CIBERSORT and MCPcounter. **(C, B)** The immune cell distribution in C1 and C2 of VTE CGs subtypes based on CIBERSORT and MCPcounter. **(E, F)** GSVA showed the pathways with significantly different distribution in C1 and C2 of SLE and VTE subtypes. Statistical significance at the level of ns ≥ 0.05, * <0.05, ** <0.01, *** <0.001 and **** <0.0001.

For preliminarily studying the correlation of CGs with immune infiltration in SLE and VTE, we identified CGs subtypes in SLE and VTE respectively by performing consensus clustering analysis. CGs subtypes of SLE divided the SLE patients of GSE61635 into C1 and C2 (**Figures 8A-C**). CGs subtypes of VTE divided the VTE patients of GSE19151 into C1 and C2 (**Figures 8D-F**). To investigate the diversities of immune characteristics between the different CGs subtypes of SLE and VTE from the cell level, the eight infiltrating immunocyte scores from MCPcounter and 27 infiltrating immunocyte percentages from CIBERSORT were compared between C1 and C2 clusters. The results suggested whether it’s the CGs subtypes of SLE or CGs subtypes of VTE, the C1 subtype presented higher infiltration levels of most immune cell populations than the C2 subtype. The C1 of SLE subtypes exhibited immune infiltration of the NK cells, T cells and CD8 T cells (**Figures 9A, B**). The C1 of VTE subtypes showed immune infiltration of the T cells, CD8 T cells, neutrophils and NK cells (**Figures 9C, D**). The GSVA algorithm was applied to calculate the enrichment score and the limma package was employed to identify the remarkably different pathways in different subtypes. The C1 of SLE subtype had higher immune activation (activation of immune response, T and B cell activation involved in immune response, myeloid leukocyte mediated immunity, etc.) than the C2 subtype (**Figure 9E**). The C1 of VTE subtype also had higher immune activation (B cell mediated immunity, somatic diversification of immunoglobulins, regulation of lymphocyte mediated immunity, positive regulation of leukocyte mediated immunity, B cell activation involved in immune response, etc.) than the C2 subtype (**Figure 9F**). Taken together, consensus clusters further demonstrated the potential interconnection between the immune infiltration landscape and CGs. The C1 cluster can be seen as an immune subtype and C2 cluster as a non-immune subtype both in SLE and VTE.

## Discussion

Venous thromboembolism (VTE) is a common disease in clinical with high mortality and misdiagnosis rate, comprising of pulmonary embolism (PTE) and deep venous thrombosis (DVT). Systemic lupus erythematosus (SLE) is a diffuse connective tissue disease mediated by autoantibodies and involving multiple systems and organs. A dysregulated immune response is recognized as a central contributor to SLE, including innate immunity and adaptive immunity (27). It has been reported that the risk of VTE in SLE patients increased significantly, and the incidence of PE and DVT in SLE patients were 12.8-fold and 19.7-fold higher respectively than the control group (28, 29). Previous studies have found the SLE disease activity index (SLEDAI) was significantly higher in SLE patients with VTE, and these patients accompanied elevated neutrophils, sensitivity C reactiveprotein (hsCRP), interleukin-6 (IL-6) and decreased complement [27]. So, there seems to be an intricate link between these two diseases. In a larger context, the coagulation process represented by VTE is closely linked to the immune response represented by SLE. In the past, Virchow’s triad of factors predisposing to thrombosis, including abnormal stasis of blood, endothelial damage, hypercoagulability, and vessel wall damage, were considered the foundation of the pathophysiology of venous thrombosis by academia (30). Nonetheless, inflammatory molecules and immune cells are now considered to play a critical role in the pathogenesis of thrombosis. Foley et al. recently reported the molecular interactions between inflammation and coagulation (31). Therefore, whether based on clinical practice or molecular mechanism research, it’s necessary to explore the evidence and significance of the potential relevance between SLE and VTE. Our research integrated the transcriptomes from the public database for the first time to reveal potential crosstalk genes between SLE and VTE and further investigated their clinical value and latent association with immune cells.

In our study, SLE and VTE gene expression matrices (GSE19151 and GSE61635) were used to identify DEGs by the ‘limma’ R package. A total of 171 crosstalk genes (CGs) were screened out by the combination of DEGs in the SLE and VTE cohorts. The results of GO and KEGG enrichment analyses present CGs mainly involved in the immune regulation and inflammatory response. Then, five hub CGs (MMP9, FOS, IGF1R, PIK3R1 and CXCL8) were identified from the PPI network of CGs. MMPs are extracellular matrix (ECM)-degrading enzymes that can involve in the inflammatory response and immune response (32). Some studies have shown that MMP-9 plays an important role in the pathogenesis of SLE by activating the inflammatory response (32, 33). Additionally, MMP-9 can degrade components of the vascular basement membrane that help inflammatory cells invade the vascular wall and induce inflammation associated with the pathogenesis of SLE, thus increasing endothelial cell permeability (34, 35). IGF1 is a member of the insulin-like growth factor family involved in mediating growth and development (36). Previous studies have shown that IGF1 was involved in immune and autoimmune diseases, including Graves’ disease and RA, and plays an anti-inflammatory role in inflammatory responses (37–39). IGF1 also exerts critical effects in endothelial-protective, anti-platelet and anti-thrombotic activities in cardiovascular disease (40). The FOS gene family consists of 4 members: FOS, FOSB, FOSL1, and FOSL2. They are found up-regulated in response to various inflammatory processes (41). Prior research suggested FOS is associated with lower indices of atherothrombotic risk in patients with cardiovascular diseases (42). Hae et al. found FOS family genes were associated with immunoglobulin A nephropathy (IgAN) and the clinical phenotypes of IgAN patients (43). The protein encoded by CXCL8 is a major mediator of the inflammatory response. CXCL8 can exert a substantial proinflammatory effect in peripheral blood mononuclear cells from SLE patients under the activation of IL-36 (44). We verified the abnormal expression of five hub CGs in different cohorts and discovered the significant correlation of infiltrating immunocytes with them according to the immune infiltration analysis. These findings are generally consistent with above-mentioned research results. In order to further study the relationship between CGs and the immune microenvironment in SLE and VTE, we respectively constructed the two subtypes for SLE and VTE in accordance with expression profiling of CGs. Both in SLE and VTE cohorts, the C1 presented higher immune cell infiltrations and stronger immune activation than the C2 subtype. Therefore, these classifications can reflect the immune landscape of SLE and VTE patients, which implied that the CGs were the important participants in the immunopathology of SLE and VTE.

Besides, we found the difference of the immune pattern between SLE and VTE according to the CIBERSORT results. Compared to the control group, multiple types of immune cells, such as neutrophils, B cells and T cells, infiltrate more in SLE samples and less in VTE samples. The results of GSVA showed the multiple immune activation-related pathways enriched in the SLE group, and the VTE group displayed the status of immunosuppression. Previous studies have shown that SLE can cause the abnormal activation of multiple immunocytes. Activated neutrophils can produce a great number of cytokines and chemokines and lead to immune dysfunction in SLE (45). It is reported that autoantibodies in vasculitis and SLE are components of neutrophils extracellular traps (NETs), a fibrous network released from the membrane of neutrophils that are activated (46). Choi et al. found that increased follicular helper-like T cells (Tfh) were positively associated with the disease activity and serum autoantibody titers *in vivo* experiments (47). The diffuse B-cell over-reactivity exhibited in SLE and plenty of autoantibodies leading to the occurrence of lupus were produced by self-reactive B cells (48). On the other hand, the antigen recognition and killing function of T cells were markedly compromised, and the functions of NK cells were significantly decreased in VTE patients (49). The decreased CD3 and CD8 levels and the increased CD4/CD8 ratio were discovered in the s acute pulmonary embolism and chronic thromboembolic pulmonary hypertension (CTEPH) patients, meaning the dysfunction of CD3+ CD8+ T Cell immunity (50).

In order to evaluate the clinical diagnostic value of CGs, lasso regression was applied to identify the best diagnostic CGs. Three biomarkers (HSP90AB1, FPR2, and RSAD2) exhibited good diagnostic capability and were validated through ROC analysis and simple factor logistic regression analysis. As an important crosstalk gene between SLE and VTE, HSP90AB1 encodes a member of the heat shock protein 90 (HSP90) family which is involved in signal transduction, protein folding, degradation and morphological evolution (51). HSP90 is an important modulator of multiple innate and adaptive inflammatory processes (52). High levels of HSP90 were discovered in the serum of SLE patients and the deposition of HSP90 in the glomerulus was found in some SLE patients (53). Besides that, the copy number variations (CNVs) of HSP90AB1 are associated with a higher risk of SLE (54). FPR2 is a member of formyl peptide-receptors (FPRs) that highly express in granulocytes, monocytes and macrophages (55). Current knowledge indicates that some genetic variants will alter FPR2 mRNA and protein expression levels and causes susceptibility to SLE (54). FPR2 also takes part in the regulation of the platelet function to promote the remission of Inflammation (56). RSAD2 (Radical S-Adenosyl Methionine Domain Containing 2) plays a role in innate immune signaling and antiviral immune response (57, 58). Some research has detected the association between RSAD2 and multiple autoimmune diseases, such as RA, SLE, and AS (59). Sezin et al. considered RSAD2 as a hub gene in the pathogenesis of SLE (60). After excluding the interference variable of multicollinearity, we constructed the VTE and SLE risk scores based on the HSP90AB1 and RSAD2 by multivariate logistic regression. Our study conducted a systematic evaluation of the performance of the two risk models, and the predictive power of the risk scores was validated in other independent cohorts using the same sequencing technique as the training cohort. The AUCs of the risk model used to predict SLE were 0.95 and 0.94 respectively in internal and external datasets. And the AUCs of the risk model used to predict VTE were 0.95 and 0.68 in the internal and external datasets. Thus, both SLE and VTE risk scores have good predictive properties for the diseases.

The present study had several limitations. Our research is based on several public cohorts from GEO datasets and detailed clinical data for most samples were missing. Of course, the data providers indicated that the baseline information of the patients, such as age, gender, and weight, was similar between the disease and the normal groups. Secondly, the crosstalk genes we identified have not been experimentally validated by further functional validation and immune correlation analysis in the current study. In addition, although we constructed risk scores and identified different disease subtypes based on CGs, further validation by prospective studies with a large sample number is needed before clinical application.

## Conclusion

We identified the three crosstalk genes (FPR2, RSAD2 and HSP90AB1) as promising diagnostic biomarkers and constructed the SLE and VTE risk models based on them respectively. Immune infiltration analysis demonstrated the intimate relevancy of CGs and immunocytes. Immune responses may play an important role in the association between SLE and VTE. Moreover, we proposed two new molecular classifications for SLE and VTE patients based on CGs, comprising immune and non-immune subtypes.

## Data availability statement

The datasets presented in this study can be found in online repositories. The names of the repository/repositories and accession number(s) can be found in the article/**Supplementary Material**.

## Ethics statement

Written informed consent was obtained from the individual(s), and minor(s)’ legal guardian/next of kin, for the publication of any potentially identifiable images or data included in this article.

## Author contributions

JFY, JY and QH designed the experiments, analyzed the data, and wrote the paper. Their contributions to the study are the same. XG and ZZ were responsible for manuscript review and providing constructive comments. All authors contributed to the article and approved the submitted version.

## Supplementary material

Supplementary Figure 1 PCA analysis of GSE61635.

Supplementary Figure 2 The correlation among hub CGs in GSE19151.

Supplementary Figure 3
**(A)** PCA analysis of VTE datasets (GSE19151 and GSE48000) before and after remove batch effect. **(B)** PCA analysis of SLE datasets (GSE61635 and GSE50772) before and after remove batch effect. **(C)** The ROC curves of the VEL and SLE combined datasets.
