# Supplementary figures and images for: Comprehensive bioinformatics analysis reveals the crosstalk genes and immune relationship between the systemic lupus erythematosus and venous thromboembolism
Source: Front Immunol. 2023 Jul 3;14:1196064. doi: 10.3389/fimmu.2023.1196064 (PMC10350530; doi:10.3389/fimmu.2023.1196064)

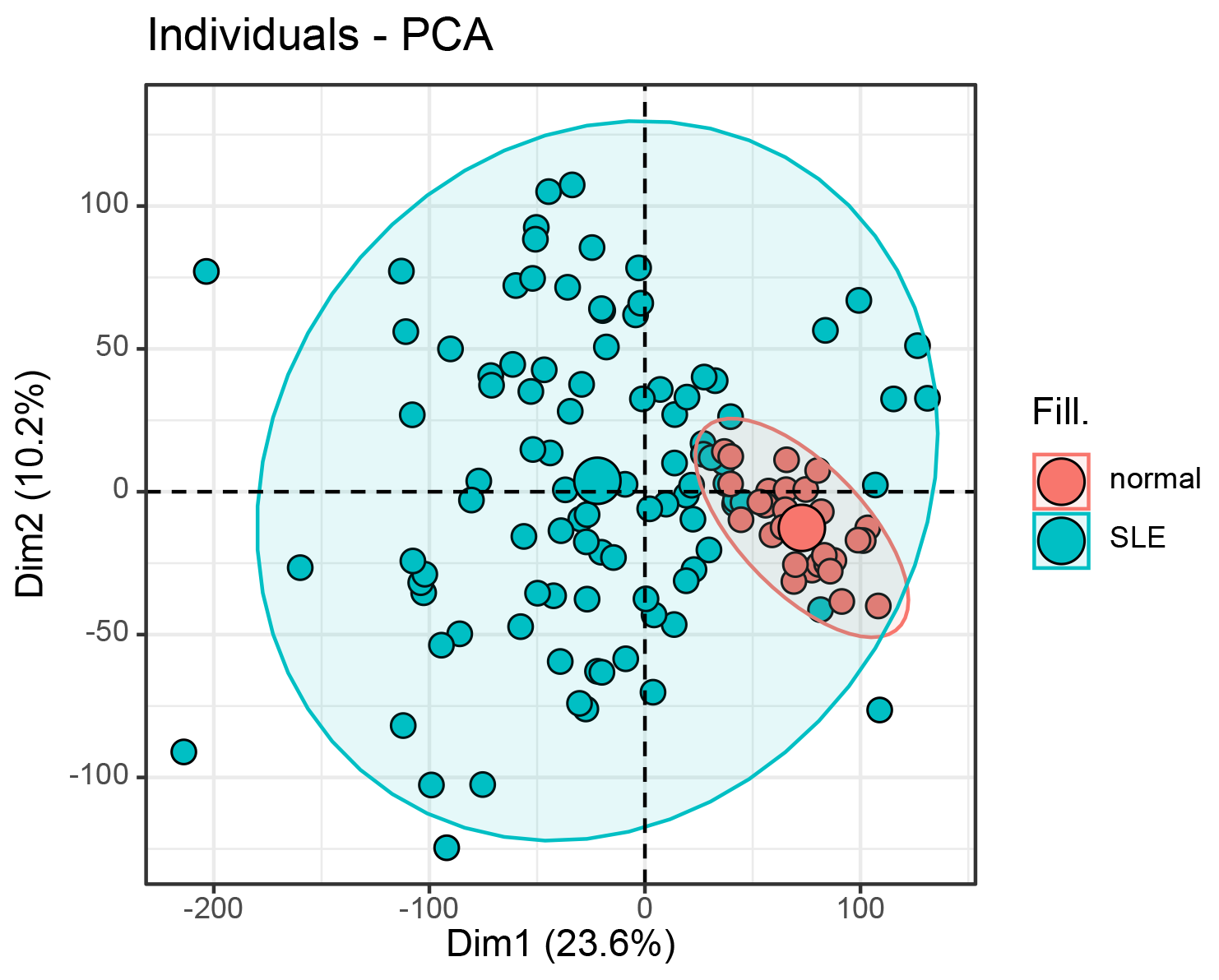

Supplement: Supplementary Figure 1 — PCA analysis of GSE61635. [file Image_1.tif]

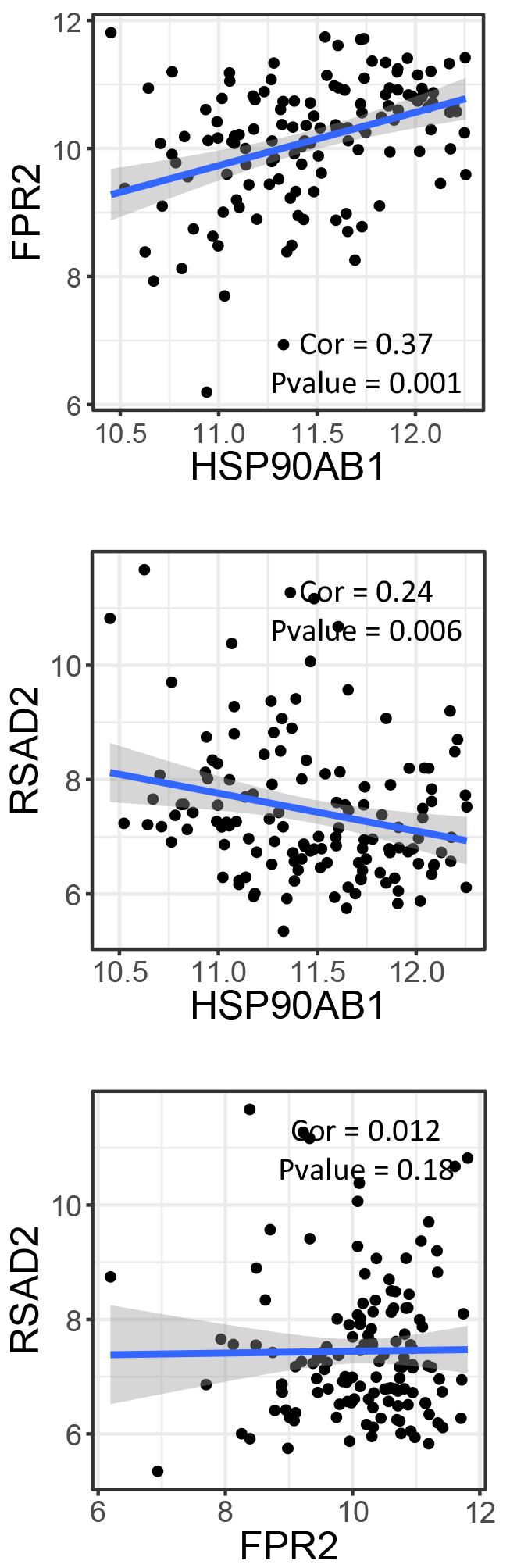

Supplement: Supplementary Figure 2 — The correlation among hub CGs in GSE19151. [file Image_2.tif]

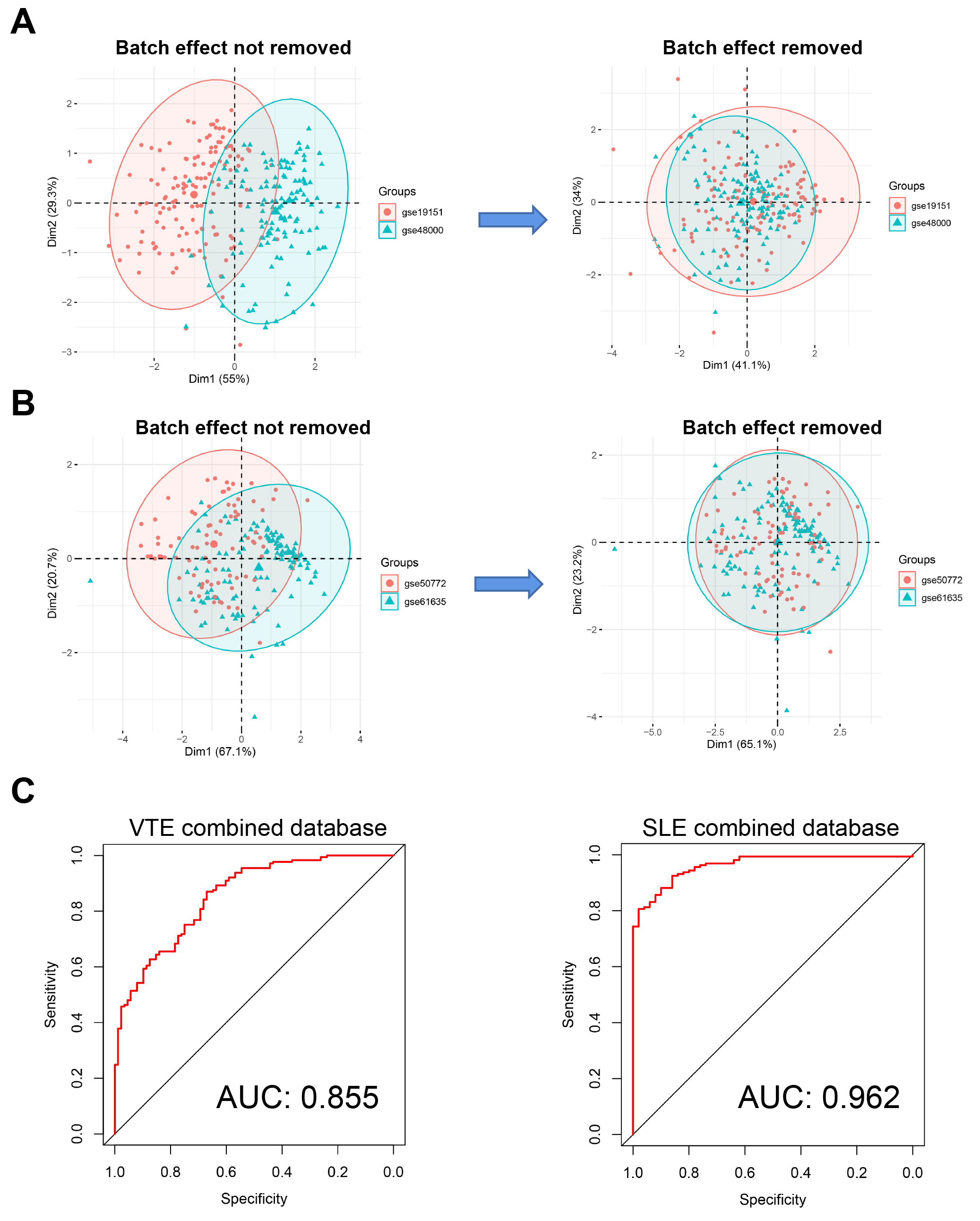

Supplement: Supplementary Figure 3 — (A) PCA analysis of VTE datasets (GSE19151 and GSE48000) before and after remove batch effect. (B) PCA analysis of SLE datasets (GSE61635 and GSE50772) before and after remove batch effect. (C) The ROC curves of the VEL and SLE combined datasets. [file Image_3.tif]
